# Buffalo Medical Library Association

**Published:** 1886-01

**Authors:** 


### Third Annual Meeting of the Buffalo Medical Library Association.

The third annual meeting of this association was held in its rooms in the Buffalo Medical College, Saturday evening, December 19th, Vice-President John Hauenstein in the chair.

The report of the Treasurer, Dr. Fredericks, showed: receipts, $275; expenses, $230; leaving a balance of $45. There is also due the Society, and which is so easily collectable as to be justly counted assets, the sum of $45. The library was moved from Dr. Samo's in April last. The Buffalo College offers the rent of the rooms free, while the expenses of an attendant, who also has charge of the White Library, is but fifty-two dollars a year. Therefore, during the coming year, all the income, except this small sum, can be devoted to the purchase and binding of periodicals, and the purchase of books.

The report of the Librarian, Dr. Mann, showed 250 bound volumes, including translations; full files of about twenty-five journals for the past four years, and many files of journals nearly complete, which, with pamphlets, number several thousand.

A fund is now available to bind many of the journals. At the meeting, pledges were made by members present to bind twelve of the journals during the coming year. He also reported the gift, by Dr. Billings, of Washington, of the "Index Catalogue of the Library of the Surgeon-General's Office."

The President desired to call the attention of the younger members of the profession to the value of the library to them. For the small sum of five dollars, they have at their command the best periodicals of this country and England, and representative ones of Germany and France. As these journals are freely circulated to the members, they may, therefore, be read not only at the library, but at their homes. The library receives about twenty-five journals, and the coming year will add several to the list. Every effort will be made to render the library as valuable as possible to subscribers.

Mention was made of the liberal subscriptions during the year to place the association free from debt.

The following are the amounts given and the names of the subscribers:

Dr. G. W. Burwell, $25.00; Dr. M. D. Mann, $25.00; Dr. John Hauenstein, $25.00; Dr. Wm. D. Granger, $15.00; Dr. Thos. Lothrop, $10.00; Dr. Conrad Diehl, $10.00; Dr. Thos. F. Rochester, $10.00; Dr. H. R. Hopkins, $10.00; Dr. Roswell Park, $10.00; Dr. Charles Cary, $10.00; Dr. Lucien Howe, $10.00; Dr. B. Folwell, $10.00; Dr. F. W. Bartlett, $5.00. Total, $175.00. The following officers were chosen for the coming year: *President*, Dr. John Hauenstein; *Vice-President*, Dr. Leon H. Harvey; *Secretary*, Dr. James W. Putnam; *Treasurer*, Dr. C. C. Fredericks; *Librarian*, Dr. M. D. Mann; *Trustees for two years*, Dr. Wm. D. Granger, Dr. Chas. G. Stockton; Drs. Lucien Howe and H. R. Hopkins holding over as trustees for another year.

Dr. G. W. Burwell, who has held the office of President since the organization of the Association, declined a re-election.

The rooms of the library are on the first floor of the Buffalo Medical College building, and are nicely furnished, making it a pleasant place to visit and a quiet place for reading. An attendant is always there from 2 to 6 p. m., and is willing and ready to wait upon members who call.

---

At the meeting of the Paris Academy of Medicine, November 3d, Jules Guerin criticized the communication of M. Pasteur, relative to the prophylaxis and cure of rabies, presented at the previous meeting, and offered the following objections:

1. There is no certainty that the disease experimented on by Pasteur is genuine rabies. Indeed, it is very certain that it is not the spontaneous rabies with which all are familiar.

2. That the induction of this artificial rabies will prevent only the effects of a succeeding inoculation with the virus of the same disease, and will not necessarily prevent true hydrophobia at all.

3. That the shepherd bitten by a mad dog might not have had rabies, even if he had not been inoculated with the virus of artificial hydrophobia, for the wounds had already been cauterized with carbolic acid, C. P.

4. That even if Pasteur's method will prevent rabies, it will not cure the disease, as has been claimed.—*L'Abeille Medicale*, November.

